# Health insurance, neighborhood income, and emergency department usage by Utah children 1996–1998

**DOI:** 10.1186/1472-6963-5-29

**Published:** 2005-04-13

**Authors:** Anthony Suruda, Thomas J Burns, Stacey Knight, J Michael Dean

**Affiliations:** 1 Intermountain Injury Control Research Center. University of Utah 615 Arapeen Drive #202 Salt Lake City, Utah 84108 USA; 2 Department of Sociology University of Oklahoma 331 Kaufman Hall Norman, Oklahoma 73019 USA

## Abstract

**Background:**

It is estimated that approximately half of emergency department (ED) usage in the U.S. and other developed countries is for non-urgent conditions and that this usage is related to availability, social, and economic factors. We examined pediatric ED usage in a U.S. state with respect to income, health insurance status, types of medical conditions, and whether introduction of managed care affected utilization by Medicaid children.

**Methods:**

Emergency department usage rates were calculated from 1996 through 1998 using Utah ED data for children with commercial health insurance, Medicaid, for uninsured children, and by income group estimating neighborhood household income from Zip code of residence. We analyzed usage following the July 1996 transition of Utah Medicaid to managed care.

**Results:**

Children with Medicaid had approximately 50% greater ED utilization rates than children with commercial health insurance or uninsured children. The majority of usage for Medicaid and uninsured children was for non-traumatic conditions. Only 35% of total ED usage was for non-emergent or non-urgent conditions and this was related to both Medicaid and low household income. Children lacking health insurance were more likely to be discharged against medical advice (OR = 2.36, 95% C.I. 1.88–2.96). There was no reduction in Medicaid ED usage following the transition to managed care.

**Conclusion:**

Usage of ED services is related to both health insurance status and income. Children lacking health insurance and Medicaid children have excessive usage for conditions which could be treated in a primary care setting. That managed care does not reduce Medicaid ED usage is consistent with findings of other studies.

## Background

The increase in utilization of emergency medical services in the U.S. and other developed countries in recent decades is related to availability of ambulatory medical services and to the provision of health insurance [1]. Acute injury [2], longevity [3], and health insurance coverage across an array of conditions [4] are related to the absolute and relative distribution of income in a society. In the United States it is estimated that from 40% to 60% of ED visits are for non-urgent conditions and that such usage is related to availability, social and economic factors, availability of other medical services, and consumer choice [5].

The Emergency Medical Treatment and Active Labor Act of 1986 mandates that hospital emergency departments provide care to anyone who needs it regardless of ability to pay. Children without health insurance have been reported to be from 1.2 to 3.7 times more likely to use ED services than children with health insurance [6,7]. A study of the 1994 National Hospital Ambulatory Medical Care Survey found a relationship between lack of health insurance and increased ED utilization by adolescents [8]. Whether the increase in ED utilization is due to lack of health insurance alone or is related to other factors is unclear. A study of 1997 U.S. data reported no relationship between health insurance status and pediatric ED utilization after controlling for covariates such as age, number of parents present in the household, ethnicity, and family income [9]. Reduction of ED utilization for non-emergent conditions has been reported following enrollment in managed care plans [10] but this has been found only in children covered by commercial health insurance and not those covered by Medicaid [11].

We analyzed whether ED usage and charges in Utah children were related to health insurance status and income. Over 90% of Utah children have some form of health insurance [12] and ED usage rates are approximately 25% less than the U.S. average [13]. Utah Medicaid changed to a state-wide managed care plan in July 1996 and we also examined whether this affected ED utilization by Medicaid children. In the Utah Medicaid program families chose from a panel of provider plans; those not choosing were assigned to a plan closest to them. No preauthorization was required for ED usage. Some of the plans had co-pay requirements for ED usage but federal regulations prohibited co-pays for Medicaid children.

## Methods

This study was approved by the University of Utah Institutional Review Board. Emergency department discharge data for all 443,040 visits for Utah children age 0–17 years for 1996 through 1998 were obtained from the Department of Health. These data excluded children admitted as inpatients through the emergency department, approximately 9% of ED visits [13] because we were unable to extract these from hospital inpatient data. Emergency department records were linked to ambulance run records for 1996–1998 using probabilistic linkage as previously described [14]. We assigned Abbreviated Injury Scale (AIS) and Injury Severity Score (ISS) scores and related information for children with injury as the principal diagnosis from the International Classification of Diseases, 9^th ^revision (ICD-9) diagnoses in hospital inpatient and emergency department records using ICDMAP-90 software from TriAnalytics, Bel Air, MD.

Health insurance status for ED visits was determined from information in emergency department records from the coding for primary payor. The various codes were assigned to the categories "Medicaid, Commercial, Managed, and Uninsured." The percentage of children lacking any health insurance for 1996 was obtained from the 1996 *Utah Health Status Survey*; the percentages for 1997 and 1998 were extrapolated from the 1996 survey and the 2000 survey [12,15]. The number of Medicaid eligible children for each year was obtained from the annual report 416 for Utah submitted to the Health Care Financing Administration. The number of children with commercial health insurance was considered to be the total number minus the number of children with Medicaid and those lacking health insurance, adjusted for the small number with other payors. Usage rates were calculated for the seven most common pediatric diagnosis-related groups (DRG) found in the data.

We purchased marketing software from Claritas Corporation (Ithica, N.Y.) which provided median household income 1996–98 based upon zip code. We assigned the values for each of these to the zip code of residence from the hospital emergency department records. We excluded hospital zip codes of residence which were outside Utah. We did not have population estimates for health insurance by zip code.

When presenting findings related to distribution of median household income we chose to examine by 'tertiles' (lowest third, middle third, and upper third) according to the income estimates for the emergency department residence data. Lower income neighborhoods were a mixture of rural and urban with a mean population density of 12,153 per square mile (median 689 per square mile). Middle income neighborhoods were urban with a mean population density of 18,566 per square mile (median 12,697 per square mile) and upper income neighborhoods were also urban with a mean population density of 24,273 per square mile (median 23,321 per square mile).

Bivariate analysis was done using the Chi Square test. Multivariate analysis was done using logistic regression. In part because the analysis data set contained more than 400,000 observations, all of the differences shown in the tables are statistically significant at the p < .05 level.

## Results

The ED usage rate per 100 children was 20.49 in 1996, 22.30 in 1997, 21.04 in 1998, and an average of 21.28 for the three year period. When analyzed by payor the majority of the increased usage in 1997 was by uninsured and Medicaid children. Children with commercial health insurance had an ED usage rate per 100 of 19.93 in 1996, 20.25 in 1997, 19.79 in 1998, and an average of 19.99. Medicaid children had an ED usage rate per 100 of 28.06 in 1996, 33.74 in 1997, 29.30 in 1998, and an average of 30.35. Uninsured children had an ED usage rate per 100 of 16.07 in 1996, 23.49 in 1997, 21.73 in 1998, and an average of 20.49.

The increased ED usage in 1997 for all groups was largely attributable to respiratory conditions such as otitis media, upper respiratory infection, and asthma. Usage for these conditions compared to 1996 rose 10% for children with commercial insurance, 35% for those with Medicaid, and 26% for uninsured children.

The seven most common DRG categories accounted for 55% of pediatric emergency visits during this period (Table 1).

**Table 1:** Emergency Department Usage for Children age 0–17 for Specific Conditions, Utah,1996–1998

	Commercial Insurance	Medicaid	Uninsured	All Children
Soft tissue trauma	3.77	3.17	3.12	3.49
Fracture or sprain of hand, forearm, or foot	1.55	0.87	0.95	1.33
Fracture or sprain of upper arm or lower leg	1.14	0.72	0.78	1.00
Concussion, < 1 hour	0.32	0.32	0.23	0.30
Otitis media or upper respiratory infecton	2.05	7.50	3.45	3.08
Gastroenteritis	1.34	2.89	2.46	1.68
Asthma or bronchitis	0.65	1.75	0.78	0.84
All conditions	19.99	30.35	20.49	21.28

Rates are per 100 children per year

There were differences with respect to age by payor and children with Medicaid had a mean age of 4.4 years, compared to children with commercial health insurance (8 years) and uninsured children (6.7 years).

Almost all of the children were discharged home (99%), 0.5% were transferred to another acute care hospital, and remainder discharged elsewhere. Children without health insurance were more likely to be discharged against medical advice (OR = 2.36, 95% CI 1.88–2.96) although the number of such discharges for the three year period was small (580, 0.1%).

For children with trauma, injury severity scores were similar for children with managed care, commercial health insurance, and no health insurance (mean, 1.8) and were somewhat less for children with Medicaid (mean, 1.6, p < .05)). Children with Medicaid had the lowest mean hospital emergency department charges of the various groups. For the common DRGs mean emergency department charges ranged from $127 to $461 (Table 2), with the average charge for all groups being $251. Overall 3.1% of emergency department records were linked to ambulance records and indicated transport by ambulance for children who were subsequently discharged from the emergency department. The majority of transports for all categories was for trauma.

**Table 2:** Emergency Department Charges for Children age 0–17, Utah, 1996–1998, for Seven Leading Diagnosis-Related Groups, All Conditions, and Payor Status

***Commercial Insurance***	*Mean Charge*	*Annual Visits*	*Average Annual Charges*
Soft tissue trauma	$245	18,360	$4,491,888
Fracture or sprain of hand, forearm, or foot	$338	7,538	$2,544,492
Fracture or sprain of upper arm or lower leg	$319	5,560	$1,775,007
Concussion, < 1 hour	$461	1,578	$726,963
Otitis media or upper respiratory infection	$148	9,993	$1,482,615
Gastroenteritis	$268	6,958	$1,864,700
Asthma or bronchitis	$220	3,173	$698,971
All conditions	$277	99,621	$27,093,422

***Medicaid***	*Mean Charge*	*Annual Visits*	*Average Annual Charges*

Soft tissue trauma	$208	3,934	$818,272
Fracture or sprain of hand, forearm, or foot	$307	1,088	$334,016
Fracture or sprain of upper arm or lower leg	$277	890	$246,530
Concussion, < 1 hour	$329	384	$126,336
Otitis media or upper respiratory infection	$127	9,314	$1.182,878
Gastroenteritis	$187	3,710	$693,770
Asthma or bronchitis	$207	2,175	$450,225
All conditions	$195	36,073	$7,376,833

***Uninsured children***	*Mean Charge*	*Annual Visits*	*Average Annual Charges*

Soft tissue trauma	$224	1,910	$427,840
Fracture or sprain of hand, forearm, or foot	$335	585	$195,975
Fracture or sprain of upper arm or lower leg	$293	481	$140,933
Concussion, < 1 hour	$416	139	$57,824
Otitis media or upper respiratory infection	$124	2,100	$260,400
Gastroenteritis	$209	975	$203,775
Asthma or bronchitis	$210	484	$101,640
All conditions	$220	11,986	$2,767,264

Estimated household annual incomes ranged from $11,250 to $94,807 based upon zip code of residence. For children who were treated in emergency departments, 22% resided in neighborhoods with the lowest tertile of household income ($11,250–$35,936), 35% resided in neighborhoods with the middle tertile household income ($36,098–49,946), and 43% resided in neighborhoods within the upper tertile of household income ($50,000 -94,807). There was an approximately two-fold variation in ED usage rates by estimated household income (Table 3).

**Table 3:** ED usage rate by estimated neighborhood household income

	1996	1997	1998
Lower income	28.4	32.6	32.1
Middle income	20.2	21.8	21.0
Upper income	16.7	17.4	15.4

Rates are per 100 children per year

Visits for emergency department care varied among the three income groups with respect to payor status (Table 4), with the proportion of uninsured children greatest in low income neighborhoods.

**Table 4:** Insurance coverage of children using emergency departments by estimated neighborhood income, Utah, 1996–1998

	Low income	Middle income	Upper income
Indemnity insurance	30.2%	37.8%	39.9%
Managed care	18.1%	31.7%	41.3%
Medicaid	40.7%	22.3%	12.7%
Uninsured	11.0%	8.2%	6.1%

The most frequent diagnoses differed by both income and health insurance status. In the lower income neighborhood, both children with Medicaid and uninsured children had otitis media as the most frequent DRG (27% and 19% respectively) while children residing in upper income neighborhoods had usage patterns that more closely resembled children with commercial health insurance than children with Medicaid. For example, the most frequent DRG for uninsured children in upper income neighborhoods was soft tissue trauma (16% of visits), as it was for children with commercial health insurance (19%).

Overall, 65% of ED visits were coded as emergent or urgent. Children whose payor was managed care were coded as "emergent" 58% of the time, followed by other types of commercial insurance and Medicaid (41% each) and uninsured children (36%).

In a multivariate model having an ED visit coded as other than emergent was related to both low income (OR = 1.30, 95% CI 1.27–1.33) and to having Medicaid (OR = 1.69, 95% CI 1.64–1.75) when age was included in the model. Using an ambulance for transport and having the ED visit coded as non-emergent was related to low income (OR = 2.14, 95% CI 1.85–2.49) but not to Medicaid (OR = 1.27, 95% CI 0.91–1.39)

## Discussion

In this study we have several important findings concerning pediatric ED visits, specific medical conditions, health insurance status, managed care, and neighborhood household income.

Emergency department usage rates during 1996–98 were approximately 50% greater for children with Medicaid than for children with commercial health insurance or uninsured children. We found a substantial proportion of pediatric ED usage was for conditions such as otitis media, upper respiratory infection, and gastroenteritis that could be expected to be treated in a lower cost primary care setting rather than in an ED (Tables 1, 2). Uninsured children and children with Medicaid had higher ED visit rates for these conditions than children with commercial health insurance and were less likely than children with commercial health insurance to be coded as "emergent." The data did not contain the time of ED visit, which together with date information would have allowed us to estimate whether ED care was sought during normal business hours when a primary care provider might ordinarily be available. The average ED charge per visit for otitis media for uninsured children, $124 (Table 2), probably exceeded the charge for initial and follow-up care from a primary care provider for the same condition. Both health insurance status and estimated neighborhood income were predictive of ED usage for these conditions which might be better treated in a primary care setting. Pediatric usage of ED services for non-urgent conditions contributes to ED overcrowding [9].

Although this study relied on secondary sources and no individual ED records were audited or reviewed, the proportion of records which were coded as non-urgent, 35%, is somewhat less than previous estimates of ED usage for non-urgent conditions [1,5] and could be due to greater participation in managed care plans than in other studies and also to exclusion of children who were admitted to the hospital from the ED.

The population under study was relatively prosperous; even the low income group's median neighborhood income was $28,000. Only 5% of neighborhoods had median estimated household incomes which were below the poverty line for a family of four, $16,000 [12]. This is consistent with reported findings that even in high per capita income countries there is an independent effect of income distribution on the health of individuals [17].

In a multivariate model we found that use of ED services for non-emergent conditions was related to both Medicaid and low income. This appears to be in contrast to the findings of Lou et al. [9] who found no relationship to income but we were unable to analyze for the additional covariates such as number of parents in the household and ethnicity which are related to family income.

Limitations of this study are the use of secondary data sources, the lack of a denominator for subcategories of children who had commercial health insurance such as managed care, and the lack of independent record review to verify the coding of ED records as emergent or urgent. The data which we examined did not contain information concerning children admitted to the hospital through the ED. The percentage of ED records which we linked to ambulance run reports was low (3.1%). The denominator used for Medicaid children was the total number of eligible children during the year rather than the average number of eligible children, and so the actual usage rates for Medicaid may be underestimated. Our analysis of income relied upon estimation of neighborhood income from zip code of residence, rather than the actual family income of any child.

Strengths of this study include the use of population-based data for an entire state and the ability to calculate separate rates for insured, uninsured, and Medicaid children. The fortuitous change of Medicaid to managed care in July 1996 allowed analysis of ED visits for Medicaid children for two succeeding years. We did not have the means to assess health outcomes following ED visits. The findings that children lacking health insurance had increasing rates of ED visits from 1996 through 1998, and that these children were more likely than others to be discharged from the ED against medical advice, suggests that lack of health insurance will be associated with adverse health outcomes.

## Conclusion

This study reports condition-specific data on ED usage which provide evidence that both economic status and health insurance status are related to pediatric emergency department usage.

ED usage for all health conditions was related to health insurance status and to estimated neighborhood income. Children in upper income neighborhoods who were uninsured had usage patterns similar to insured children, while uninsured children in low income neighborhoods had usage patterns similar to Medicaid children. Both the middle and upper income neighborhoods in this study were in urban areas where walk-in urgent care centers provided an alternative source of care during weekends and evening hours. This is consistent with our finding that, regardless of insurance status, children living in high income areas were less likely to have ED visits coded as other than "emergent or urgent" than children living in low income areas.

We found that the July 1996 transition of Medicaid to managed care had little effect on Medicaid usage in 1997 and 1998. There was actually a small increase in following the transition. This is consistent with findings in other states [11]. The transition to Medicaid had no disincentives for ED usage and so we would not expect to find an effect of managed care unless there was increased access to primary care providers for children. Whether ED usage for minor health conditions is due to lack of timely access to a provider, consumer habits, or lack of requirement for a financial co-payment [16] for ED use is unknown. The lack of effect is unfortunate in that the largest single category of expenditure for annual ED charges in Medicaid children in this study was $1,182,878 for otitis media and upper respiratory infection, conditions which should be less expensive to treat in a primary care setting.

## Abbreviations

DRG Diagnosis-Related Groups

ED Emergency Department

OR Odds Ratio

CI Confidence Interval

## Competing interests

The author(s) declare that they have no competing interests.

## Authors' contributions

All authors participated in the study design, preliminary data analysis, and writing the manuscript. Anthony Suruda was the principal investigator and completed the data analysis. Thomas J. Burns contributed the analysis by estimated income. Stacey Knight provided statistical support. J. Michael Dean facilitated acquisition and analysis of the data.

## Pre-publication history

The pre-publication history for this paper can be accessed here:


